# Polyploidy and Myc Proto-Oncogenes Promote Stress Adaptation via Epigenetic Plasticity and Gene Regulatory Network Rewiring

**DOI:** 10.3390/ijms23179691

**Published:** 2022-08-26

**Authors:** Olga V. Anatskaya, Alexander E. Vinogradov

**Affiliations:** Institute of Cytology, Russian Academy of Sciences, 194064 St. Petersburg, Russia

**Keywords:** polyploidy, epigenetic regulation, *Myc*, chromatin opening, adaptation to stress, gene regulatory network, cancer, cardiovascular diseases, neurodegeneration, hypertranscription

## Abstract

Polyploid cells demonstrate biological plasticity and stress adaptation in evolution; development; and pathologies, including cardiovascular diseases, neurodegeneration, and cancer. The nature of ploidy-related advantages is still not completely understood. Here, we summarize the literature on molecular mechanisms underlying ploidy-related adaptive features. Polyploidy can regulate gene expression via chromatin opening, reawakening ancient evolutionary programs of embryonality. Chromatin opening switches on genes with bivalent chromatin domains that promote adaptation via rapid induction in response to signals of stress or morphogenesis. Therefore, stress-associated polyploidy can activate Myc proto-oncogenes, which further promote chromatin opening. Moreover, Myc proto-oncogenes can trigger polyploidization de novo and accelerate genome accumulation in already polyploid cells. As a result of these cooperative effects, polyploidy can increase the ability of cells to search for adaptive states of cellular programs through gene regulatory network rewiring. This ability is manifested in epigenetic plasticity associated with traits of stemness, unicellularity, flexible energy metabolism, and a complex system of DNA damage protection, combining primitive error-prone unicellular repair pathways, advanced error-free multicellular repair pathways, and DNA damage-buffering ability. These three features can be considered important components of the increased adaptability of polyploid cells. The evidence presented here contribute to the understanding of the nature of stress resistance associated with ploidy and may be useful in the development of new methods for the prevention and treatment of cardiovascular and oncological diseases.

## 1. Introduction

Somatic polyploidy exists in tissues of nearly all multicellular organisms, including higher and lower plants, invertebrates, and vertebrates [1,2,3,4,5]. In humans and mammals, polyploidy may be a part of normal developmental programs and may result from stress caused by a variety of pathological conditions. In the normal mammalian development, cell polyploidization accompanies the early postnatal organogenesis of the neocortex, neuroglia, heart, retina, blood vessels, blood, liver, skin, placenta, kidneys, and other organs [4,6,7,8,9]. In humans, polyploidy develops mostly in the heart, where almost every normal cardiomyocyte contains 4–16 genomes [10]. Pathological conditions that enhance the accumulation of genomes in cells include neurodegenerative disorders, cardiovascular diseases, diabetes, wound healing, etc. [8,11,12,13,14,15,16,17,18,19,20]. Recent studied have indicated that polyploidy promotes cancer initiation, progression, metastasis, and drug resistance [21,22,23,24]. The main features of polyploidy include the association with stress and the ability to enhance stress adaptation under normal and pathological conditions [25,26]. The data confirming the relationships between polyploidy and stress under normal conditions only recently appeared, significantly contributing to the understanding of the biological role of genome duplications. It has become clear that in the development of mammals, the accumulation of genomes in somatic cells coincides with critical periods of postnatal growth when cells combine proliferation and differentiation and are subjected to stress associated with global physiological rearrangements [11,12,14,27,28]. For example, developmental cardiomyocyte polyploidization coincides with metabolic rearrangements accompanied by the transition to the microenvironment, with high oxygen content associated with DNA damage response, oxidative stress, and a decrease in expression of nuclear lamina filament lamin B2 (Lmnb2), which regulates nuclear breakdown prior to cell division [29,30]. Accordingly, in macrophages, polyploidization induced by inflammation is triggered by replication stress and DNA damage response [31]. The data obtained by single-cell sequencing of human tetraploid cell lines provide clear evidence that these cells undergo high rates of DNA damage during DNA replication in the first S phase following induction of tetraploidy [32]. In pathological conditions, polyploidy is associated with and aids in survival when faced with different types of stress. Thus, in cardiovascular diseases, including tetralogy of Fallot, cardiomyopathy, hypertension, and ischemic heart disease, polyploidy helps to cope with hypoxia and mechanical tension [10,33,34,35,36,37,38]. In neurodegeneration, polyploidy promotes adaptation to oxidative damage, ischemia, and inflammation [8]. In wound healing and myocardial infarction, genome accumulation stimulates adaptation to DNA damage, mechanical stress, and inflammation [9,34]. Moreover, in polyploid giant cancer cells, multiple genomes confer resistance to chemo- and radiotherapy [23,39,40,41,42,43,44,45,46,47]. These data are in agreement with previous observations of plant polyploid cells, indicating that multiple genomes can live under conditions under which diploid cells do not survive [25]. 

Notwithstanding the benefits under stress, polyploid cells cannot outcompete their diploid counterparts under normal conditions because of their slower proliferation, DNA instability, high energetic cost, and mitotic defects [17,25,48,49]. The ability of various types of polyploid cells to survive under extremely stressful conditions despite multiple detrimental effects suggests that genome accumulation creates new phenotypic features via specific pathways of gene regulation. Polyploidy does not alter gene dosage balance. Therefore, it cannot exert strong effects on the expression of separate genes. Furthermore, a tight association with stress suggests that polyploidy can operate via epigenetic changes. 

Ploidy-associated epigenetic regulation has attracted increasing attention because it enables genome reorganization and cell fate change in adverse environments and in response to extreme stress [39,41,42,50]. Moreover, this mode of regulation operating via ploidy-related epigenetic rearrangements is characteristic of carcinogenesis and resistance to therapy [42,43]. In this review, we extensively analyze the literature, which indicates that polyploidy provokes global genome reorganization via chromatin relaxation induced by stress-related Myc overexpression and ploidy-related architectural rearrangements. The relaxation is observed at both high and low levels of chromatin organization. We also provide evidence that polyploidy activates global transcription amplifiers belonging to Myc-family oncogenes, which further promote chromatin opening.

## 2. Stress-Induced Myc Promotes Polyploidy and Vice Versa

Oncogenes of the Myc family (c-Myc and N-myc) are the most powerful and well-studied stress-response-related amplifiers of global transcription [51,52,53]. Recent studies indicate that, on the one hand, polyploidy is associated with Myc upregulation and, on the other hand, that Myc can promote polyploidy. Many features associated with polyploidy are also manifestations of activated Myc. Below, we outline the literature providing evidence that polyploidy can upregulate Myc and vice versa and that overexpressed Myc and polyploidy have many common manifestations. 

### 2.1. Overexpressed Myc Induces Polyploidy

Stress-related Myc overexpression can trigger and enhance polyploidy. Supraphysiological Myc expression is sufficient to trigger polyploidy in cells with various mitotic potentials. Myc induces DNA replication in quiescent, terminally differentiated cells, including mammalian cardiomyocytes, neurons, kidney cells, and post-mitotic epithelial cells of Drosophila [8,54]. Myc enforces genome accumulation in normal cells with weak mitotic capacity (hepatocytes, neurons, megakaryocytes, keratinocytes, and trophoblast cells) [55,56] and in cycling polyploid cancer cells of various origins [43,45,57,58,59,60]. Moreover, N-Myc upregulation was recently documented in a wide variety of cancer cells with polyploid genomes [61]. Overexpressed Myc promotes polyploidy and endoreduplication via the induction of S-phase regulators, such as cyclins of CCNE and CCND families, cyclin-dependent kinases CDK2 and CDK4, and transcription factors of the E2F family [62,63,64,65]. Myc also interacts with DNA replication origins and activates them epigenetically through histone modifications and nucleosome remodeling, promoting re-replication in specific loci and chromosomal regions by acting as an illegitimate replication-licensing factor [65,66]. 

Surprisingly, Myc-associated S-phase enforcement is not accompanied by the acceleration of mitotic cellular division [52,58,67]. Recent observations indicate that blocking of mitotic progression can occur as a result of Myc-induced DNA instability, disturbance of mitotic spindle geometry, and metaphase and anaphase duration [64,68]. Another reason for the link between overexpressed Myc and polyploidy is that the latter can abrogate G1 and G2 checkpoints controlling DNA damage and DNA replication [52,57,58,65]. 

### 2.2. Polyploidy Upregulates C-Myc 

**Figure 1:**
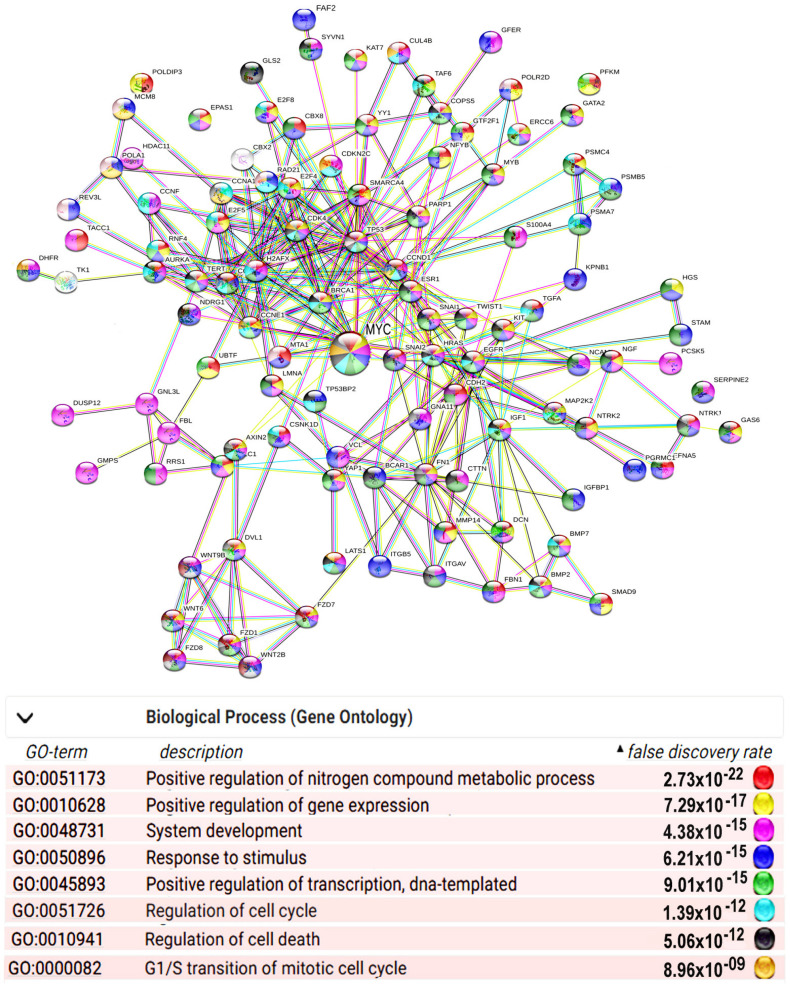
The most connected component of protein interaction networks of significantly ploidy- induced genes in the c-Myc interactome of human and mouse heart, liver, and placenta. The network was constructed using the String server (https://string-db.org/, assessed on 5 May 2022). The data for network construction were taken from [72]. Color coding reflects the Biological Process of Gene Ontology (GO) database. The gene symbols containing portions of various colors indicate that a gene is involved in several GO biological processes. The fraction of a circle that is a particular color does not convey any meaning; the circle is simply divided into a number of partitions to reflect the number of GO processes involved.

In normal cells, polyploidy is associated with the induction of Myc and its interactants in human and mouse heart, liver, and placenta [16,43,55,69,70,71,72,73] (Figure 1). Endopolyploidy and Myc are coupled in polyploid cells of *Drosophila* arising in development and wound healing [74,75,76]. Polyploidy also upregulates Myc in cancer cells. For example, in high-grade diffuse large B-cell lymphoma, drug-induced and drug-resistant polyploid cells also show c-Myc overexpression [77]. A similar finding was reported with respect to polyploid melanoma cells generated by paclitaxel [58]. These cells were found to overexpress c-Myc and reduce the expression of MAD2, an essential component of the molecular core of the spindle assembly checkpoint (SAC), indicating impairment of this checkpoint [58]. An extensive bioinformatics study performed with 10,000 primary human cancer samples and essentiality data from 600 cancer cell lines provided evidence that polyploidy in cancer is associated with induction of N-myc [61] (Supplementary Table S1 from [61]). Accordingly, the authors revealed the steady upregulation of c-Myc targets (Figure 1E in [61]), which was not accompanied by c-Myc induction (Supplementary Table S1 in [61]), confirming that chromatin opening can enhance the efficiency of amplifiers, even if their expression is not changed or is even decreased. The authors observed the induction of gene modules related to DNA repair, proliferation, and cell cycle, as well as the downregulation of gene modules involved in immune response and allograft rejection [61]. These data confirm that polyploidy is associated with the upregulation of c-Myc in various conditions.

Thus, Myc and polyploidy respond to similar stimuli, including a wide variety of stressful and pathological conditions. Overexpressed Myc promotes polyploidy via the stimulation of rapid S-phase entry, DNA replication, and mitotic spindle abnormalities, whereas polyploidy activates Myc through stress response, genetic instability, and replicative stress associated with chromatin opening [64,78,79]. 

## 3. Myc and Polyploidy Increase Stress Resistance

It is well-established that Myc enhances stress tolerance to various environmental clues. Myc promotes protection from hypoxia, oxidative stress, drugs, and DNA instability [80,81,82]. Moreover, Myc confers cells with resistance to apoptosis. An association between overexpressed Myc and protection from apoptosis was observed in tumors of various origins [77,83,84,85,86]. Another important protective feature of Myc is that when overexpressed, it enables tumor cells to deregulate their microenvironment and evade the host immune response [82]. 

Polyploidy is also associated with stress and protects cells from hypoxic, hyperoxic, and genotoxic environments and increases resistance to drugs [39,40,44,46,57,80,81,87]. Furthermore, polyploidy protects cells from aging related stress. This association has been well-established in cells of insects and mammals. For example, the increased polyploidization was discovered in spermathecal glands of honeybee queens during senescence, accompanied by genotoxic and oxidative stress [88]. The authors accounted for this phenomenon with a selective repression or induction of gene expression [88]. In mammals, the tight association between polyploidy and senescence was documented in neurons, hepatocytes, vascular smooth muscle cells, and even in cancer cells [16,43,47,89,90]. Importantly, polyploidy can also safeguard cell survival under energy depletion. For example, the giant polyploid nuclei originating from nuclear and cellular fusion arise in the insect vectors of Chagas disease, especially under starving-stress conditions [91]. The epithelial cells of Malpighian tubes of blood-sucking insects demonstrate polyploidization via nucleus fusion after 4.5 month of starvation [92,93]. 

Polyploidy mitigates consequences of DNA instability via the upregulation of pathways related to DNA damage response and DNA repair. This connection was well-established based on mRNA sequencing data from roughly 10,000 primary human cancer samples and essentiality data from approximately 600 cancer cell lines [61]. The link between polyploidy, DNA instability, and related pathways was also indicated for giant polyploid cancer cells [40,94,95], tumor-initiating cells in vivo, RPE1 cell in culture [32], and yeast [96]. Polyploidy also confers resistance to apoptosis. This feature was documented for polyploid cancer cells, giant cancer cells, and polyploid cells of normal tissues [40,61,72,97]. Another unexpected protective feature of polyploidy that is also common with Myc is immune evasion. The data of experimental studies and extensive transcriptome analyses indicate that in thousands of tumors and in normal tissues, polyploidy is associated with the downregulation of biological pathways and markers related to immunity [61,71,72,77,98]. Thus, ploidy-associated protection from stress confers polyploid cells with the ability to survive under conditions that are not suitable for diploid cells. An important cause of particular stress resistance of polyploid cells is the association between polyploidy and overexpressed Myc. On the one hand, stress-induced Myc can trigger polyploidy; on the other hand, polyploidy can upregulate Myc. Thus, there is a link between polyploidy, overexpressed Myc, and stress resistance.

## 4. Myc and Polyploidy Promote Chromatin Opening 

### 4.1. Myc Promotes Chromatin Repositioning from the Nuclear Periphery to the Inner Part

Data obtained from B cells indicate that c-Myc can accelerate chromatin decondensation via its repositioning from the nuclear periphery to the inner part and by promoting a nuclear architectural shift from long-range to short-range contacts, leading to a near doubling of loops and topologically associated domains (TADs) [64,99]. Data recently derived from skeletal muscle stem cells provide evidence that Myc can regulate TAD composition and structure via TAD splitting, merging, rebuilding, rearranging, or disappearing [99,100]. Thus, overexpressed Myc can relax chromatin at the periphery of polyploid cells.

### 4.2. Polyploidy Stimulates Chromatin Transition from the Outer Part to the Inner Part of the Nucleus

**Figure 2:**
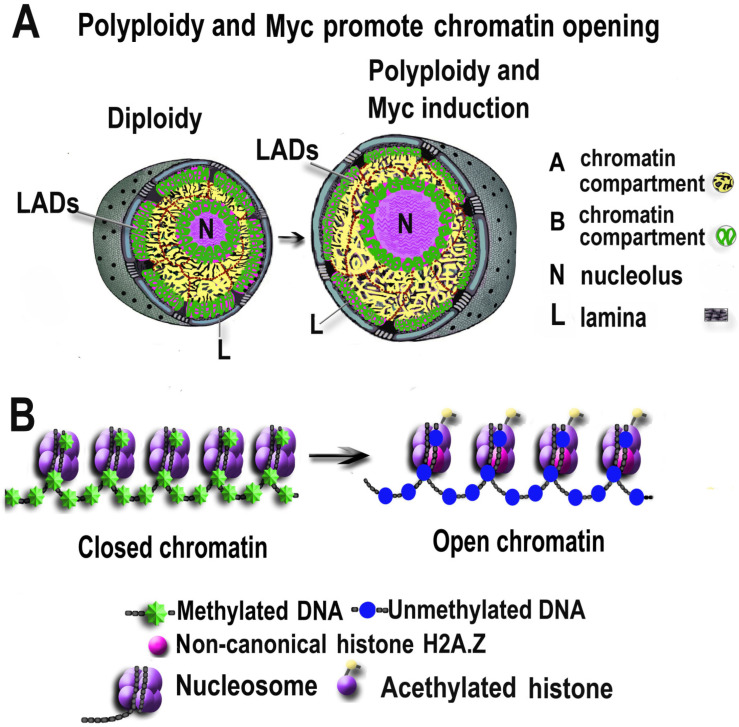
Polyploidy and overexpressed Myc promote chromatin opening via common effects at high (**A**) and low (**B**) levels of organization. A-chromatin opening due to lamina-associated domain detachment from the lamina and chromatin transition from B (closed) to A (open) state. B-chromatin opening due to DNA hypomethylation, histone acetylation, and substitution of canonical histones with non-canonical histone H2A.Z.

Polyploidy can also potentially boost the effect of overexpressed Myc related to chromatin remodeling. It is well-established that genome duplication decreases the nucleus surface-to-volume ratio, which suggests the ability to affect high levels of chromatin organization [101,102]. Initial evidence was recently presented showing that this decrease affects the architecture of chromatin located at the periphery of the nucleus. Hi-C data obtained from KBM7 cells indicate that genome accumulation leads to preferential loss of nuclear lamina (NL) interactions of lamina-associated domains (LADs) [101,102]. The loss occurs as a result of increased competition for NL contacts originating from the decrease in nucleus surface-to-volume ratio [101,102]. The LADs exhibit heterochromatic features, including low gene density, low transcriptional activity, and late replication timing [103]. In the LADs detached from the lamina, some “repressed LAD promoters” became active as a result of their removal from the LAD context and transitioned to the nuclear interior [104]. This transition increases the number of compartments with open A-chromatin at the expense of compartments with closed B-chromatin [104]. B-to-A chromatin compartmental transition is a universal mechanism of topological activation of gene expression in development and differentiation [105]. For example, ploidy-related gene activation via large-scale chromatin topology rearrangement operates in cardiogenesis. Chromatin regions transitioning from B to A are strongly enriched for heart developmental genes upregulated during human and mouse cardiogenesis and in cardiac differentiation of hPSCs [105]. Accordingly, data obtained from synthetic autotetraploid plants (watermelon, soybean, and Arabidopsis) also indicate that polyploidy shifts the A-to-B chromatin balance toward the actively transcribed A-chromatin [106,107]. Moreover, recent studies provide evidence that genome duplications reorganize topologically associated domains (TAD) packaged within A- and B-chromatin compartments via the increase in intra-TAD interaction and reorganization of chromatin loops [106,108]. In addition, polyploidy in giant cancer cells can enlarge chromosome territories [109]. The ability of polyploidy to relax chromatin via its repositioning from the periphery to the center of the nucleus, as well as to alter LADs and TADs geometry, is also characteristic of cancer cells [109,110]. Thus, both c-Myc and polyploidy can reposition chromatin from the nuclear periphery to the inner part of the nucleus, promoting its relaxation (Figure 2).

## 5. Myc and Polyploidy Open Chromatin at the Low Level of Organization and Activate Transcription

### 5.1. Myc Opens Chromatin and Reinforces Expression of Already Actively Transcribed Genes via Binding of E Boxes

Myc proteins bind enhancer-box (E-box)-containing CACGTC sites [111]. E-box-containing genes can be divided into genes with “high-affinity E boxes” and “low-affinity E boxes” [65,112]. Researchers discovered that the two classes of E boxes can be differentiated by a marked enrichment of CpG islands and open chromatin marks, including DNA hypomethylation and histone modifications [65,113]. Consistently with this observation, Myc target genes were reported to demonstrate higher basal expression (even in the absence of Myc) relative to non-target genes [112]. Thus, Myc promotes transcription by further reinforcing the expression of already intensely working genes and that of genes with bivalent chromatin marks (i.e., genes containing both active and silent chromatin H3K4me3 and H3K27me3 marks) [15,51,73,114,115]. This phenomenon explains why overexpressed Myc shows different faces in various tissues [52,80,116]. Because the set of actively transcribed genes varies depending on the cell type, manifestations of Myc overexpression differ according to cell type. This feature indicates that Myc is not a specific transcription factor but a general amplifier that increases RNA content [117,118].

### 5.2. Myc Activates Chromatin via the Induction of Pol I, II, and III

Myc oncogenes can also activate chromatin via the induction of Pol I-, II-, and III-transcribing rRNA, tRNA, and mRNA [119,120]. Pol I, II, and III locally open the double-stranded DNA so that one strand of the exposed nucleotides can be used as a template for the RNA synthesis [119,121]. The effects of MYC on genes transcribed by all three polymerases are mostly inducing [111,122,123]. Myc activates genes participating in growth, proliferation, cell cycle (G1/S transition), energy metabolism, purine biosynthesis, ribogenesis, protein turnover, and other pathways, whereas Myc-repressed genes include cyclin-dependent kinase inhibitors and genes involved in apoptosis and adhesion [111,124]. Owing to these properties, Myc oncogenes can reverse DNA-damage-induced proliferation arrest and activate processes related to adaptation, wound healing, and tumorigenesis [75,125,126]. Almost all of the effects of Myc on the expression of specific target genes are weak and are often below the twofold threshold for expression difference, even when Myc levels are manipulated to increase several orders of magnitude [111,125,126]. 

### 5.3. Myc Interacts with Chromatin-Remodeling Partners

Myc-related facilitation of transcription can also be executed via the interaction with chromatin-remodeling partners. Myc isoforms interact with transformation/transcription-domain-associated protein (TRRAP), which is a scaffold protein of several large protein complexes involved in chromatin remodeling [127]. TRRAP also cooperates with p400 chromatin-remodeling helicase, which is the ATP-hydrolyzing subunit of the chromatin-remodeling Tip60/Ep400 complex that substitutes the canonical histone H2A for histone H2A.Z [128]. Histone H2A.Z increases enhancer activity, facilitating the binding of transcription factors and chromatin remodelers to trigger transcription and changes in the 3D chromatin structure [129]. H2A.Z histone also plays an important role in chromosome segregation and cell cycle progression, whereby H2A.Z upregulates the expression of key cell cycle genes, such as c-Myc, Myc-N, KIi67, and AURKA [130]. In addition, H2A.Z fine tunes processes of cell renewal and mediates the establishment of bivalent promoters of developmental genes during embryonic stem cell differentiation [131]. The reduction in H2A.Z in mESCs results in loss of pluripotency, premature differentiation, and senescence [130]. Another important Myc partner implicated in chromatin opening is WDR5, which facilitates histone H3 Lys4 (H3K4) methylation and increases the affinity of MYC for active promoters [65]. Myc can also be involved in chromatin activation via interaction with INI1 (SMARCB1/hSNF5/BAF47), a core member of the SWItch/sucrose non-fermentable (SWI/SNF) chromatin remodeling complex [132]. It was also shown that the basic helix–loop–helix region associated with Myc interacts with INI1 repeat 1 (Rpt1) in ATP-dependent chromatin organization and transactivation [53].

Thus, the literature provides evidence that Myc overexpression can promote chromatin opening via the repositioning from the surface to the periphery of the nucleus (high level of chromatin organization) and via interaction with E boxes, RNA polymerases, and chromatin remodelers (low level). Because polyploidy can open chromatin, we speculate that when Myc overexpression coincides with polyploidy, their effects on chromatin can be cooperative. Therefore, it is reasonable to suggest that overexpressed c-Myc and polyploidy can cause prominent chromatin decompaction, leading to global activation of transcription and alteration of gene regulatory networks and cell fate decision. The abundance, diversity, and evolutionary conservatism of biological processes and functions that are coregulated by polyploidy and Myc indicate that they are responsible for transcriptional regulation via global epigenetic changes.

### 5.4. Polyploidy Promotes DNA Hypomethylation, Histone Modification, and Substitution of Canonical Histones with Non-Canonical Histones

Ploidy-associated chromatin activation and opening at high levels of organization induce a coherent response at lower levels. Genome duplications promote DNA hypomethylation and an increase in the amount of various open chromatin marks activating gene expression. For example, an study performed with colorectal cancer cells (line LS174T) showed that the onset of tetraploidy is associated with the demethylation of non-mobile pericentromeric repetitive elements SST1 and transposable elements LINE-1, comprising approximately 17% of the entire genome [133]. In line with these results, data obtained from ovarian cancer cells show a strong statistical association between polyploidy, increasing quantitative LINE1 DNA hypomethylation, and hypomethylation of centromeric DNAs Chr1 and Sat2 [134]. LINE-1 methylation is usually considered a marker of genome methylation [133]. Therefore, the association between polyploidy and hypomethylation of LINE-1 suggests that genome duplication decreases DNA methylation and opens chromatin in a substantial part of the genome. The concomitance of polyploidy and global hypomethylation was also found in trophoblast giant cells and mouse embryonic fibroblasts [135,136]. In concordance, data obtained from plants, including *Medicago truncatula* symbiotic nodule cells, neotetraploid rice, Arabidopsis, and soybean, reveal causal relationships between polyploidy and chromatin opening, mostly via DNA hypomethylation and/or demethylation of DNA packaging protein histone H3 (specifically the H3K27me3 mark) [137,138]. Polyploidy was also found to activate histone acetylation in human embryonic kidney cells and in bread wheat *Triticum aestivum L* [139,140]. In addition to DNA hypomethylation, histone acetylation, and histone demethylation, polyploidy can promote the substitution of canonical histones with non-canonical histone H2AZ, which is necessary for chromatin relaxation [135,141]. Thus, Myc and polyploidy can promote chromatin opening and activation of transcription at various levels of organization.

## 6. Common Biological Effects of Overexpressed Myc and Polyploidy Manifest in Embryonic Phenotype and Metabostemness

Chromatin opening affects basic biological processes and cell fate. In multicellular organisms, open chromatin is a feature of undifferentiated pluripotent stem cells that maintain plasticity in biological regulation, specific metabolic state, and dual capacity to self-renew and differentiate into all cell types [142,143,144,145]. In differentiated cells, open chromatin awakens silent genes, enabling sensing of multiple, simultaneous, and often opposing signals (e.g., adult and fetal) in the environment [142,145]. In addition, chromatin opening causes dedifferentiation and activates programs of embryonality and pluripotency [142]. These features are acquired via the remodeling of chromatin at various levels of organization, including nuclear periphery, TADs, LADs, posttranslational modifications of histones, and DNA methylation [143,144,146]. Polyploidy and overexpressed Myc are associated with many manifestations related to chromatin opening. Importantly, most of these features relate to pluripotency.

### 6.1. Myc and Polyploidy Activate Programs of Embryonality

Myc oncogenes activate signaling pathways involved in pluripotency and embryogenesis (including NOTCH, BMP, TGFb, PI3K, HIPPO, and WNT), as well as pathways of epithelial to mesenchymal transition and cell cycle progression, contributing to self-renewal through maintenance of undifferentiated states [83,147,148]. Moreover, Myc is one of the four Yamanaka factors that reprogram differentiated cells to an embryonic-like state [148]. A polyploid state, as well as the Myc oncogene, can maintain stemness. The activation of pluripotency programs was observed in polyploid giant cancer cells (PGCCs) of ovarian, breast, prostate, and colorectal cancers, as well as Burkitt’s lymphoma [39,44,45,46,60,95,149,150,151,152,153,154,155]. Moreover, in these cells, polyploidy was accompanied by the expression of the key embryonic stem cell markers Oct4/Nanog, Sox2, SCF, and c-kit, as well as markers of cancer stem cells (CD44 and CD133) [156,157]. In addition, endocycling cells of *Drosophila* epidermis and mouse cornea endothelial cells that participate in wound healing promote stemness through the activation of the Hippo pathway [75]. Researchers suggest that wound-induced polyploidization enables tissue repair when cell division is not a viable option [19,75,158]. Data recently obtained via mRNA sequencing of human and mouse heart, liver, and placenta and from isolated hepatocytes and cardiac interstitial cells provide evidence that polyploidy is associated with the induction of signaling pathways related to stress response, growth, G1/S transition, and multipotency, including NOTCH, BMP, TGFb, PI3K, HIPPO, and WNT, as well as epithelial-to-mesenchymal transition [2,48,71,72,73,159,160]. Thus, both, Myc and polyploidy can enhance programs of stemness.

### 6.2. Myc and Polyploidy Upregulate Genes with Bivalent Promoters

Another common feature of polyploidy and Myc is the upregulation of bivalent genes [73,114,161]. These genes harbor two opposite epigenetic modifications of histone H3, the repressing H3K27me3 mark and the activating H3K4me3 mark, in their promoters or enhancers, [162]. Bivalent genes are poised for transcription and are capable of rapid activation [162]. Prevalent in embryonic stem cells, bivalency is postulated to poise/prime lineage-controlling developmental genes for rapid activation during embryogenesis while maintaining a transcriptionally repressed state in the absence of activation cues [163]. This is particularly important for key developmental genes and enhancers, the activation of which in a short time window during differentiation may be crucial [164]. 

The activation of genes with bivalent chromatin was also observed in pathologies associated with cell polyploidization and manifestations of stemness, including cardiovascular diseases and cancer [73,162,165,166]. Moreover, the tight link between polyploidy, bivalent genes, and programs of embryonality seems to be a fundamental and evolutionarily conserved phenomenon. Thus, human ohnologs (genes retained in duplicates after whole-genome duplications) are most strongly enriched in the bivalent genes and genes implicated in development, showing an analogy with somatic polyploidy [16]. The biased retention of expression-regulating and developmental genes can be explained by their particular importance indicated by the strong purifying selection on them [167,168].

### 6.3. Myc and Polyploidy Are Associated with Metabostemness and Hypertranscription

It is well-known that Myc induces metabolic modification with features of metabostemness observed in enhanced glycolysis, glutaminolysis, ribosome biogenesis, and hypertranscription, which collectively enable cells to acquire continuous energy supply via reserved energy-producing pathways [9,111,169,170,171,172,173]. Additional sources of metabolites serve as essential cofactors for epigenetic enzymes regulating DNA methylation, posttranslational modifications of histones, and nucleosome position [174,175]. 

Metabolic pathways of polyploid cells also demonstrate manifestations of stemness [11,71,72]. Similarly to pluripotent stem cells, polyploid cells can simultaneously derive energy from pathways that are incompatible with differentiated cells, promoting adaptation to stress associated with energy deprivation [49,72,176,177,178,179,180,181,182]. Recent data obtained from polyploid cells from normal tissues of animals and plants, various tumors, and yeasts indicate that these cells possess energetically flexible metabolism combining glycolysis glutaminolysis and oxidative phosphorylation [43,57,72,183,184,185]. Thus, in addition to stem cells, polyploid cells can adjust their energy metabolism to the environment, which is impossible for differentiated cells.

Under hypoxia, lack of nutrients, starvation, or severe genotoxic stress, polyploid cells can be in a state of dormancy with predominantly glycolytic energy supply, which is a feature of metabostemness [40,43,46,88,92,93]. Under normoxia, polyploidy is usually accompanied by enhanced glycolysis, glutaminolysis, hypertranscription, active protein synthesis, and ribosome biogenesis, which are also manifestations of embryonality [2,6,71,79,186,187]. All these features of metabostemness have also been described in endopolyploid cells of *Drosophila* in development and wound healing, in cancer polyploid cells, and in cells of normal mammalian tissues [7,57,178,184,188]. 

### 6.4. Myc and Polyploidy Awaken Programs of Unicellularity

According to the ancient origin of Myc, it can be traced to unicellular organisms using deep phylostratigraphy [189], and several Myc-associated traits are also characteristic of unicellular organisms. For example, as in unicellular organisms, Myc activates glycolysis, glutaminolysis, ribogenesis, and features of epithelial-to-mesenchymal transition, confirming the association between Myc and the reactivation of evolutionary ancient gene modules [82,190,191]. Recent phylostratigraphic data indicate that both polyploidy and Myc shift the expression of genes toward unicellularity [43,73]. Polyploidy-associated features of stemness and metabostemness are also observed in unicellular primitive organisms, further confirming that polyploidy promotes dedifferentiation and primitive ancient traits, including the activation of ancestral gene modules related to glycolysis, epithelial-to-mesenchymal transition, housekeeping genes, cell cycle, ribosome biogenesis, and flexible adaptive reaction to stress [22,190,191,192,193,194,195,196]. The connection between polyploidy and unicellularity is not surprising because polyploidy is an ancient phenomenon that appeared together with reproductive cysts of unicellular organisms [197]. Moreover, Vladimir Niculescu considers polyploidization to be part of the already reactivated unicellular programs and acquired unicellular lifestyle [196,197]. Recent data obtained via phylostratigraphy indicate that in human and mouse heart and liver, polyploidy shifts the evolutionary age balance of the expressed genes from the late metazoan phylostrata toward the upregulation of unicellular and early metazoan phylostrata [73]. It has been shown that the human interactome consists of unicellular and multicellular giant clusters [167,192]. In cancer cells, the expression of the unicellular cluster is enhanced, whereas the multicellular cluster is suppressed [167,198]. Accordingly, in polyploid cancer cells, the expression of the unicellular cluster is upregulated, whereas the multicellular cluster is downregulated, even compared with diploid cells of the same cancer, indicating that polyploidization of cancer cells enhances their unicellular properties [16,199]. The tight connection between cancer polyploidy and unicellularity confirms the atavistic theory of oncogenesis, which suggests that cancer is a reversal from a multicellular to a unicellular state [200,201,202,203].

## 7. Myc and Polyploidy Are Possibly Evolutionarily Conserved Partners Increasing Adaptation to Stress via Epigenetic Plasticity, Metabolism, and DNA Damage Protection

### 7.1. Myc Increases the Ability of Polyploid Cells to Outcompete Diploid Cells under Stressful Conditions

Polyploidy and overexpressed Myc appear in response to physiological and pathological stress. Both exert similar effects on chromatin architecture and many biological processes, including chromatin relaxation, phenotype plasticity, stemness, metabolic rearrangements, regulation of gene expression, and adaptation. Myc is a sensor of intrinsic and extrinsic stress belonging to early response master regulators that reacts to a wide variety of stimuli [204]. In response to stress, Myc promotes adaptive reactions, increasing cell resistance and flexibility. Under long-term and severe activation, Myc can also cause unwanted effects, including DNA instability, hypertranscription, replicative stress, and cell cycle disturbance [65]. These flaws can stimulate polyploidy, which also exerts beneficial and detrimental effects [62]. On the one hand, polyploidy increases stress resistance and adaptability (similarly to Myc), and on the other hand, it promotes genetic instability and chromatin relaxation (also similarly to Myc), contributing to further Myc induction [43,57,71,72]. Thus, it is reasonable to suggest that Myc and polyploidy can reinforce each other, facilitating the manifestation of both adaptive and adverse effects. Likely as a result of this duality, polyploid cells acquire competitive advantages only under stressful conditions [25].

### 7.2. Epigenetic Phenotypic Plasticity, Energy Reserve, and Protection from DNA Instability Boosted by Myc Might Help Polyploid Cells to Adapt

**Figure 3:**
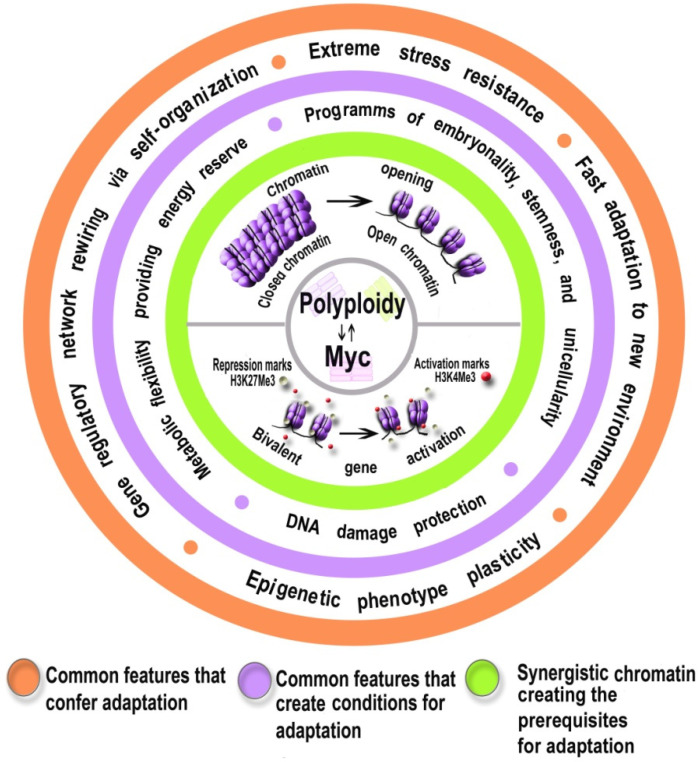
The most important common features of polyploidy and overexpressed Myc that promote resistance to extreme stress and confer the ability to rapidly adapt to new environments.

Why might the Myc–polyploidy partnership enable particular adaptation to extreme stress? Data obtained from therapy-resistant giant polyploid cancer cells (GPCCs) suggest that the polyploid-related ability to “survive at the brink” [43] originates from three main sources. First, stress-related polyploidy can promote rapid adaptation to changing environments via global genome reorganization (also termed ‘genome chaos’), leading to high epigenetic and phenotypic plasticity and dedifferentiation or stemness [41,43,46,205,206]. Secondly, it protects cells from genomic instability [25,40,57,69]. Thirdly, it provides enhanced energy supply via additional pathways of ATP and HADH production [49,72,185] (Figure 3). Epigenetic phenotypic plasticity, stemness, and genome reorganization can be caused by chromatin opening, DNA damage, and bivalent gene induction promoting the ability for rapid transitions from one state to another via network self-organization and adaptive search for an energetically favorable attractor state [41,44,206,207,208,209,210,211,212,213]. Ploidy-associated cell protection from DNA instability is provided by additional genomes buffering DNA damage and by an efficient but error-prone DNA repair system, which is a characteristic of unicellular organisms [214,215]. Additional energy is provided by the activation of pathways of glycolysis, glutaminolysis, and NADH production, as well as by the induction of Warburg and Crabtree effects [72,183,184,185]. All three features enabling rapid identification of attractors are also related to ancient, unicellular organisms, confirming a tight link between polyploidy and recapitulation of evolutionarily ancient programs [200,201]. Thus, dedifferentiated state, error-prone DNA repair, and a particular metabolic patterns with enhanced Warburg and Crabtree effects were previously well-established in yeast, amoeba, and other organisms [183,197].

In humans, polyploidy, in partnership with Myc, may help cells to survive under pathologic conditions associated with various diseases, including hypertension, congenital heart diseases, neurodegeneration, inflammation, and even cancer [8,14,17,43]. In the case of cancer, polyploidy confers particular resistance to therapy and drugs. Therefore, it is of particular importance to identify effective therapies that target polyploid cancer cells. These three ploidy-specific features are promising targets for therapy directed at the elimination of polyploid cells, particularly in cancer. Specifically, therapy can be directed toward the correction of metabolism (via impairment of glycolysis or glutaminolysis), the weakening of stemness through the changing of epigenetic state, and the removal of DNA instability protection by blocking of error-prone primitive DNA repair pathways. It is also tempting to suggest that Myc silencing could be effective. However, such an approach is controversial. On the one hand, Myc silencing can be beneficial, as in endopolyploid cells, it causes depolyploidization, leading to a reduction in cell size and ploidy [216]; on the other hand, Myc silencing can be detrimental because silent Myc can cause deep-cell dormancy accompanied by severe metabolic deprivation and permanent therapy resistance [217]. Therefore, more data and knowledge are needed to come to conclusion about Myc-silencing therapy for polyploidy targeting.

## 8. Conclusions

Polyploid cells demonstrate particular plasticity and adaptation to stress. On the one hand, this feature is beneficial because it helps to adapt to various pathological states, including cardiovascular diseases, neurodegeneration, inflammation, wound healing, and regeneration. On the other hand, this feature is detrimental because it promotes carcinogenesis, metastasis, and cancer relapse caused by cancer therapy resistance. Currently, the nature of ploidy-related adaptability is not completely understood. Here, we highlight literature data indicating that polyploidy can regulate gene expression via extensive epigenetic changes promoting chromatin relaxation. Owing to increased nuclear volume in polyploid cells, the decreased surface-to-volume ratio results in partial detachment of LADs from the nuclear lamina, thereby changing the structure of LADs and TADs and increasing the proportion of euchromatin. Polyploidy also promotes DNA hypomethylation and chromatin modifications, relaxing chromatin. Altogether, these changes awaken bivalent genes that are rapidly activated in response to stress or signals of morphogenesis. Contributing to the opening of chromatin, polyploidy also activates global transcription amplifier oncogenes of the Myc family, which, like polyploidy, contribute to the opening of chromatin. Myc oncogenes can also accelerate the accumulation of genomes, enhancing polyploidization. As a result of these cooperative effects of polyploidy and activated Myc, genetic instability occurs, which, together with chromatin opening and induction of bivalent genes, can result in genomic chaos, increasing epigenetic and phenotypic plasticity and the ability to search for adaptive states of cellular programs through gene regulatory network rewiring. Ploidy-related regulatory and phenotypic plasticity is manifested in (1) traits of stemness, dedifferentiation, and unicellularity; (2) flexible energy metabolism; and (3) effective protection from genome instability consisting via buffering of DNA damage and mutation effects and in complex DNA repair that combines primitive unicellular error-prone repair pathways and advanced multicellular error-free repair pathways. We suggest that these three features are important components of the increased adaptability of polyploid cells. The presented evidence can be useful in the development of new types of therapy with the aim of eliminating polyploid cancer cells. The evidence presented herein could also be useful for the development of new measures of preventive medicine with the aim of preventing excessive polyploidization of cardiomyocytes and neurons in order to reduce the risk of cardiovascular and neurodegenerative diseases.
